# Supplementary material for: JQ-1 ameliorates schistosomiasis liver granuloma in mice by suppressing male and female reproductive systems and egg development of Schistosoma japonicum
Source: PLoS Negl Trop Dis. 2022 Aug 9;16(8):e0010661. doi: 10.1371/journal.pntd.0010661 (PMC9362908; doi:10.1371/journal.pntd.0010661)
Supplement: S1 Table — (DOCX) [file pntd.0010661.s001.docx]

| **Genes** | **Forward primers** | **Reverse primers** |
| --- | --- | --- |
| SjPSMD4 | ACTTTGAACAGGAGATGGCGA | GCCTCAGGACAACGGAACC |
| SjPlk1 | ATCCGCTCGCTTCTACA | TCAACCGCAGCACCTAC |
| SjNanos1 | GAATTCTTTGTCTCCTGGAAATGCCTGC | AAGCTTGCCTGGGCAGTATTTGAT |
| IL-1β | CTGAACTCAACTGTGAAATGC | AATCCATGTGCGCCCGACAACC |
| IL-6 | ACCACGGCCTTCCCTACTT | GGGCTTTGGCACAGTCCTCATTA |
| IL-13 | CTTGCTTGCCTTGGTGGTCT | CTTGCACATTGTAGCTGTGTACC |
| IL-17 | ACCGCAATGAAGACCCTGAT | CTCACCAACAAAGGCGTAGA |
| IL-21 | GCCAGATCGCCTCCTGATTA | GGCTTTGGAGGTGCCACGATAG |
| IL-22 | CATGCAGGAGGTGGTACCTT | ATGTGGCGGAAGTAGTTCCC |
| TNF-α | TTGCTCTGTGAAGGGAATGG | AGGACATCAGGGGATGCTGT |
| TGF-β | ACAATTCCTGGCGTTACCTT | CATCAAGGCACCAGGATCAGT |
| IFN-γ | GGTCAACAACCCACAGGTCC | gggagtgcagtaatggctt |
| GAPDH | TTCACCACCATGGAGAAGGC | GGCATGGACTGTGGTCATGA |

Supplementary Table. Sequences of Quantitative PCR primers
