# Supplementary material for: JQ-1 ameliorates schistosomiasis liver granuloma in mice by suppressing male and female reproductive systems and egg development of Schistosoma japonicum
Source: PLoS Negl Trop Dis. 2022 Aug 9;16(8):e0010661. doi: 10.1371/journal.pntd.0010661 (PMC9362908; doi:10.1371/journal.pntd.0010661)
Supplement: Attachment — Submitted filename: Response to reviews for plos ntd1(1)CLEAN.docx [file pntd.0010661.s002.docx]

Editor

*PLOS Neglected Tropical Diseases*

June 13, 2022

Dear Editor:

We would like to thank the editors of *PLOS Neglected Tropical Diseases* for giving us the opportunity to revise our manuscript PNTD-D-22-00330, originally titled “JQ-1 ameliorates schistosomiasis liver granuloma in mice by suppressing male and female reproductive systems and egg development of *Schistosoma japonicum*.” We also thank the reviewers for their careful reading and insightful comments on our previous draft. We have taken their comments into consideration in preparing our revised manuscript, which has resulted in a paper that is clearer, more compelling, and more comprehensive. The following letter provides our point-by-point responses to the editor’s and reviewers’ comments.

Best regards,

Dr. Liu

Corresponding Author

**Dear reviewers #1 and #2**:

We are grateful for your comments on our manuscript. We have amended the relevant parts in the revised manuscript according to your suggestions. In addition, some of your questions are answered below.

**Reviewer #1:**

The author studied the effect of JQ-1 on reproductive systems and egg development of Schistosoma japonicum to ameliorates liver granuloma in mice. The authors detected the male-female pairing rate, egg production, reproductive system and the expression of related genes of Schistosoma japonicum after JQ-1 treatment in vivo and in vitro. The data suggested JQ-1 treatment ameliorated S. japonicum egg–induced hepatic granuloma and suppressing the development of both the male and female reproductive systems and female egg production. This manuscript be of interest. However, there are some concerns about this paper.

*Major concerns*

1. JQ-1 is the inhibitor of BET. The mRNA and protein expression levels of BET protein family should be detected in this study. Authors may provide such data in different groups with or without JQ-1 treatment.

**Response:** Thank you for the suggestion. JQ-1, as a first-in-class potent and selective inhibitor of the BET signaling pathway, has been widely used in biology studies. JQ-1 interacts with the BRD pocket in a manner competitive with acetylated peptide binding, resulting in the displacement of BET proteins from acetylated chromatin in cells exposed to this inhibitor and disruption of the associated transcript initiation and elongation factors. Thus, JQ-1 may not affect the mRNA and protein expression levels of BET protein family members. Although the BET protein family contains four members (BRD2, BRD3, BRD4, and BRDT), only BRD2 is present in *Schistosoma japonicum*. In our study, the levels of BRD2 mRNA have now been detected, and the results have been provided in the revised manuscript.

2. Please include some positive controls for JQ-1 treatment in in vivo and in vitro models.

**Response:** Thank you for this suggestion. The bromo- and extra-terminal domain (BET) signaling pathway plays an important role in cell proliferation, immune responses, and pro-inflammatory events. Since the first BET inhibitor, JQ-1, was developed in 2010, it has been widely used as a potent and selective inhibitor of the BET signaling pathway in biology studies. However, no suitable positive control drug for JQ-1 has been reported to date. Thus, in our study, JQ-1 was used as a pharmacological tool for elucidating the roles and functions of BET in the reproductive system development of *S. japonicum*.

3. No negative control group (normal control) in In vitro experiment

**Response:** A negative control group was used in our experiment. Numerous previous studies (including ours) have shown that DMSO (0.1%) has no effect on egg production or the reproductive system development of *S. japonicum*. Thus, for the sake of brevity, the results of the negative control group in the in vitro experiment were not provided in the manuscript. In response to the reviewer’s comment, however, we provide some of those results below for convenience:

Fig 1. Effect of JQ-1 on egg production in *S. japonicum*.

ov

ov

t

t

Fig 2. Effects of JQ-1 on the testes and ovary of *S. japonicum*.

4. Effect of JQ-1 on cell apoptosis in male-female pairs of S. japonicum should be included.

**Response:** For the sake of brevity, we chose not to report the effect of JQ-1 on cell apoptosis because previous work (including ours) has shown that JQ-1 has no effect on cell apoptosis. For convenience, we provide two references below:

Ding N, Hah N, Yu RT, Sherman MH, Benner C, Leblanc M, He M, Liddle C, Downes M, Evans RM. BRD4 is a novel therapeutic target for liver fibrosis. Proc Natl Acad Sci U S A. 2015 Dec 22;112(51):15713-8.

Ding H, Yang X, Tian J, Wang X, Ji Y, El-Ashram S, Ren C, Shen J, Liu M. JQ-1 ameliorates schistosomiasis liver fibrosis by suppressing JAK2 and STAT3 activation. Biomed Pharmacother. 2021 Dec;144:112281.

5.	Why did the author treat the S. japonicum infected mice with JQ-1 after four weeks’ infection? Please provide the reasons in the manuscripts.

**Response:** Thank you for your good question. *S. japonicum* lays eggs 4 weeks after infection. The aim of our present work was to investigate whether JQ-1 could attenuate *S. japonicum* egg–induced liver granuloma by suppressing the male and female reproductive systems and egg development of these parasites. Therefore, mice in the experimental group were injected intraperitoneally with JQ-1 daily for 15 days of successive administration starting 4 weeks after infection.

6.	It would be nice to detect the levels of SjNanos1, SjPlk1 mRNA in vivo.

**Response:** We agree with the reviewer’s good suggestion. Thus, we detected the levels of *SjNanos1* and *SjPlk1* mRNA in vivo and included those results in the revised manuscript.

7.	JQ-1 has also been used as a pharmacological tool for elucidating the roles and functions of BET in mammals. How to evaluate the effect of JQ-1 on host mice? Do JQ-1 has the toxic effects on mice, especially hepatotoxicity? And how about the expression of inflammatory factors in mice treated with JQ-1 alone.

**Response:** Thank you for your good questions. Since JQ-1 was developed in 2010, its conventional dose and course of treatment in mouse experiments has been 50 mg/kg of body weight per day for approximately 15 days. To our knowledge, there are no reports of obvious toxic effects or hepatotoxicity. However, JQ-1 has been shown to have promising anti-inflammatory effects through its ability to interfere with pro-inflammatory transcription factors, such as NFκB, STAT3, STAT5, and RORC. Thus, inflammatory factor expression was inhibited in mice treated with JQ-1 alone.

8.	The method of egg count in liver tissue is not clear. Because eggs deposited in liver are not evenly in schistosomiasis mice, liver tissues should be taken in the fix sites in each mouse in control group and test group.

**Response:** Thank you for this good comment. As the reviewer said, we did not clearly describe how we counted the eggs in the liver. In fact, liver tissue was collected from the same site of each mouse to count the eggs. We clarified this in the Methods section of the revised manuscript.

9.	The Figures problems:1) Illustrations for Fig 3 and Fig 4 were misplaced; 2) Fig 4, the labeled texts on axes in graphs are blurred and indistinct. 3) Fig5: the present graphs are unreasonable; the text labeled on axes are not accurate. 4）Fig7: graphs should be combined. 5) the morphological photos in Fig2,3,4,8,9 should be marked with test groups in figures and provided more illustration details.

**Response:** We are grateful for the reviewer’s comment and have modified all Figures in the revised manuscript.

*Minor errors:*

10.	The English of the manuscript needs to be carefully edited.

**Response:** The manuscript has been professionally edited by an editor at Onboard Editing whose preferred language is English. The editor has a Ph.D. in pharmacology, an expertise in neuroscience, and 9 years of full-time medical and science writing/editing experience, including for top-tier medical journals.

11.	P1 line15-16: Schistosomiasis is a serious and widespread parasitic disease caused by infection with Schistosoma japonicum._

12.	Revised：…….by infection with Schistosoma.

**Response:** Thank you for pointing out this error. We have revised the manuscript accordingly.

13.	P2 line38-39: schistosomiasis is a serious disease caused by infection with the parasite Schistosomiasis japonicum.

14.	Revised：schistosomiasis japonicum is a serious disease caused by infection with the parasite S. japonicum.

**Response:** Thank you for pointing this out. We revised our typo in the genus name. However, we believe that the first part of the original statement is accurate, that is, schistosomiasis is the name of the disease.

15.	P3 line58: S. japonicum. — Use italics and check the whole text especially in reference part.

**Response:** Thank you for pointing out this typographical issue. We have checked for this throughout the entire text of the revised manuscript, including the Reference list.

16.	P4 line74: Schistosoma japonicum — revised: S. japonicum. and check the whole text.

**Response:** Thank you for pointing out our typographical errors. We have revised the manuscript accordingly.

**Reviewer #2:**

In this study, the effects of JQ-1, a selective inhibitor of BET protein family, on adult worm development of Schistosoma japonicum, especially reproductive system development, were investigated. The results showed that JQ-1 could reduce the egg laying and the germ cell division in the adult worms in vitro and in vivo, which resulted in the decrease of egg granuloma formation and release of proinflammatory factors in liver of the infected mice. However, when considering the use of JQ-1 as an anti-schistosomiasis drug, the authors do not answer or ignore two important questions:

1.	The fate of eggs treated with JQ-1, that is, whether they can still be excreted from the intestine.

**Response:** Thank you for this good comment. Eggs deposited by *S. japonicum* adult female worms are the chief cause of tissue damage consisting of hepatic granuloma and fibrosis. In our study, we wanted to confirm that JQ-1 ameliorates schistosomiasis liver granuloma in mice by suppressing the male and female reproductive systems and egg development of *S. japonicum*. Thus in this study, we detected the fate of the eggs in the liver, but not in the feces.

2.	Whether or not JQ-1 has the effects on host germ cells? Otherwise, it is not practical or reasonable to evaluate the pharmacological effect of JQ-1 on Schistosoma japonicum reproduction. Although the authors also observed that JQ-1 inhibited the expression of reproductive genes SjPlk1and SjNanos1 in the worms, they did not discuss the relationship between these effects and the known influence of JQ-1 on the function of mammalian BET proteins.

**Response:** The bromodomain and extra-terminal (BET) family contains four members (bromodomain motif-containing proteins BRD2, BRD3, BRD4, and BRDT). JQ-1 treatment produces a reversible contraceptive effect by targeting the activity of BRDT in male germ cells of mice, validating BRDT as a male contraceptive target.

In our study, JQ-1 was used solely as a pharmacological tool for elucidating the roles and functions of BET in suppressing the development of the male and female reproductive systems in *S. japonicum*. Of the four BET family members, only BRD2 is present in *S. japonicum*. The 75% amino acid difference between human BRD2 and *S. japonicum* BRD2 may provide a target for selective inhibitor design for *S. japonicum*, which warrants further study as a new approach for the treatment or prevention of schistosomiasis.
